# Spontaneous splenic rupture in a patient with factor XIII deficiency: A case report

**DOI:** 10.1016/j.radcr.2025.06.026

**Published:** 2025-06-27

**Authors:** José M. Zepeda Torres, Essaú Brambila López, Laura B. Alegría López, Isac I. Ramírez Preciado, Derek G. Cantú Ochoa, Heriberto González Canal, Héctor A. Benítez Jauregui, Maria H. García Ramírez, Daniel A. Ángel Montoya, Carolina Topete Rodríguez

**Affiliations:** General Surgery, High Specialty Medical Unit, Specialty Hospital, National Western Medical Center, Mexican Social Security Institute, Guadalajara, Mexico

**Keywords:** Spontaneous splenic rupture, Factor XIII deficiency, Coagulation disorder

## Abstract

Spontaneous splenic rupture is a rare, life-threatening event, infrequently associated with congenital factor XIII deficiency, a rare coagulation disorder. We present a case of an 18-year-old female with known factor XIII deficiency who presented with acute abdominal pain and was diagnosed with spontaneous splenic rupture. The patient underwent splenectomy due to hemodynamic instability and massive hemorrhage. Postoperative management included factor XIII replacement and standard postsplenectomy prophylaxis. Spontaneous splenic rupture in the setting of factor XIII deficiency is exceedingly rare, with only a limited number of cases reported in the literature. This case highlights the importance of considering coagulation disorders in the differential diagnosis of atraumatic splenic rupture. Prompt diagnosis and multidisciplinary management, including surgical intervention when necessary, are critical for optimizing patient outcomes. This case underscores that factor XIII deficiency can lead to spontaneous life-threatening hemorrhage. Clinicians should maintain a high index of suspicion for this pathology in patients with known coagulopathies, and timely intervention is essential to improve patient outcomes. The importance of this approach is to quickly treat it when needed because is very deadly.

## Introduction

Spontaneous (atraumatic) rupture of the spleen is an uncommon but potentially life-threatening event. It accounts for only about 3% of all splenic rupture cases and carries a significant mortality risk (approximately 12% in reported series). Typically, splenic rupture is associated with trauma; however, in rare instances it occurs without any history of it, necessitating a high index of suspicion for alternative causes. Patients often present with acute abdominal pain (which may radiate to the left shoulder as Kehr’s sign), along with nonspecific symptoms such as nausea, abdominal distension, or signs of hypovolemic shock in advanced cases. In over 90% of atraumatic splenic ruptures, an underlying predisposing pathology can be identified [1].

Among potential risk factors, congenital coagulation disorders represent an especially rare precipitant of spontaneous splenic rupture. In fact, Factor XIII deficiency, along with prothrombin deficiency, are among the rarest of inherited coagulation factor deficiencies. Factor XIII deficiency is characterized by a frequency of 1 in 2,000,000 in the general population and can lead to severe bleeding manifestations. In particular, hemophilia A (factor VIII deficiency) has been documented to cause spontaneous intra-abdominal hemorrhage, including at least 1 neonatal case of splenic rupture in a hemophiliac infant. Factor XIII deficiency, an inherited clotting disorder that leads to unstable fibrin clot formation, is far less common than hemophilia and has been previously reported as a cause of spontaneous splenic rupture in the literature [2]. While rare, spontaneous splenic rupture has been reported in the setting of Factor XIII deficiency. Moreover, to the best of our knowledge, recurrent spontaneous splenic rupture in a patient with Factor XIII deficiency has been documented in only few cases [3,4]. Here, we present a case of spontaneous splenic rupture in an adolescent with known factor XIII deficiency. This case highlights the clinical significance of spontaneous splenic rupture in the setting of a congenital coagulation disorder and reviews relevant literature on its pathophysiology, management, and outcomes.

## Case presentation

An 18-year-old female with a known history of factor XIII deficiency (congenital quantitative Factor XIII deficiency) presented to the emergency department with acute abdominal pain. The pain began suddenly in the left upper quadrant (LUQ) while she was performing routine household activities. It was intense and persistent, and she noted no history of trauma to the area. Her medical history was significant for factor XIII deficiency managed jointly with hematology, and she had chronic severe anemia (World Health Organization grade III) related to her condition. There was no personal or family history of hemophilia or other bleeding disorders aside from the factor XIII deficiency, and she was not on any anticoagulant medications.

**Fig. 1:**
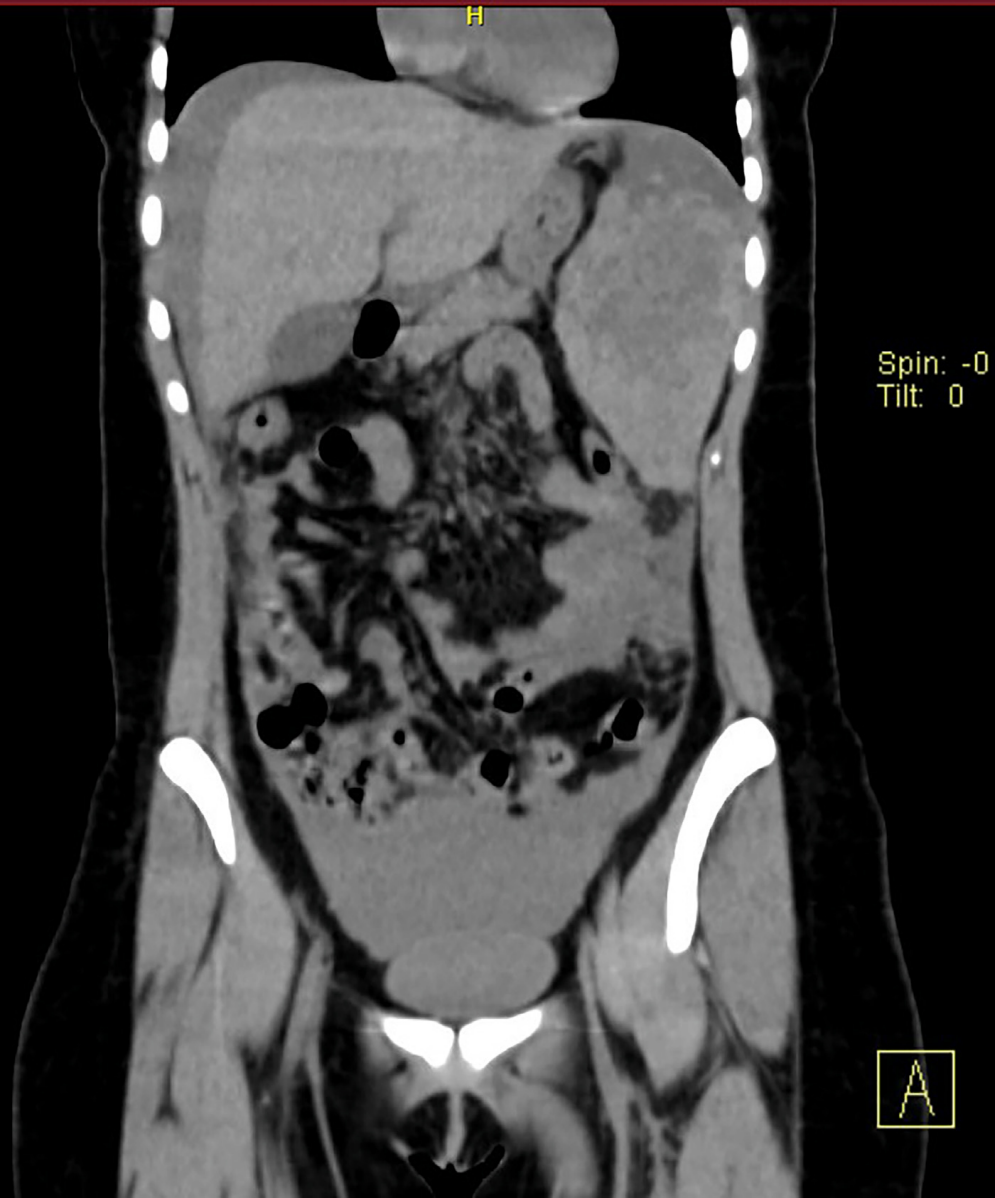
Coronal view. Changes in morphology and density are confirmed at the level of the spleen, which is observed surrounded by a large hematoma reaching 144 × 74 × 154 mm in its maximum diameters, with a calculated volume of approximately 853 mm, there is no contrast phase, however with window modification it suggests capsular origin, this hematoma displaces the splenic flexure of the colon inferiorly, it is associated with free fluid that extends from the phrenic spaces to the pelvic hollow, this high-density fluid reaches densities ranging from 44 to 60 Uh and is associated with hemoperitoneum.

**Fig. 2:**
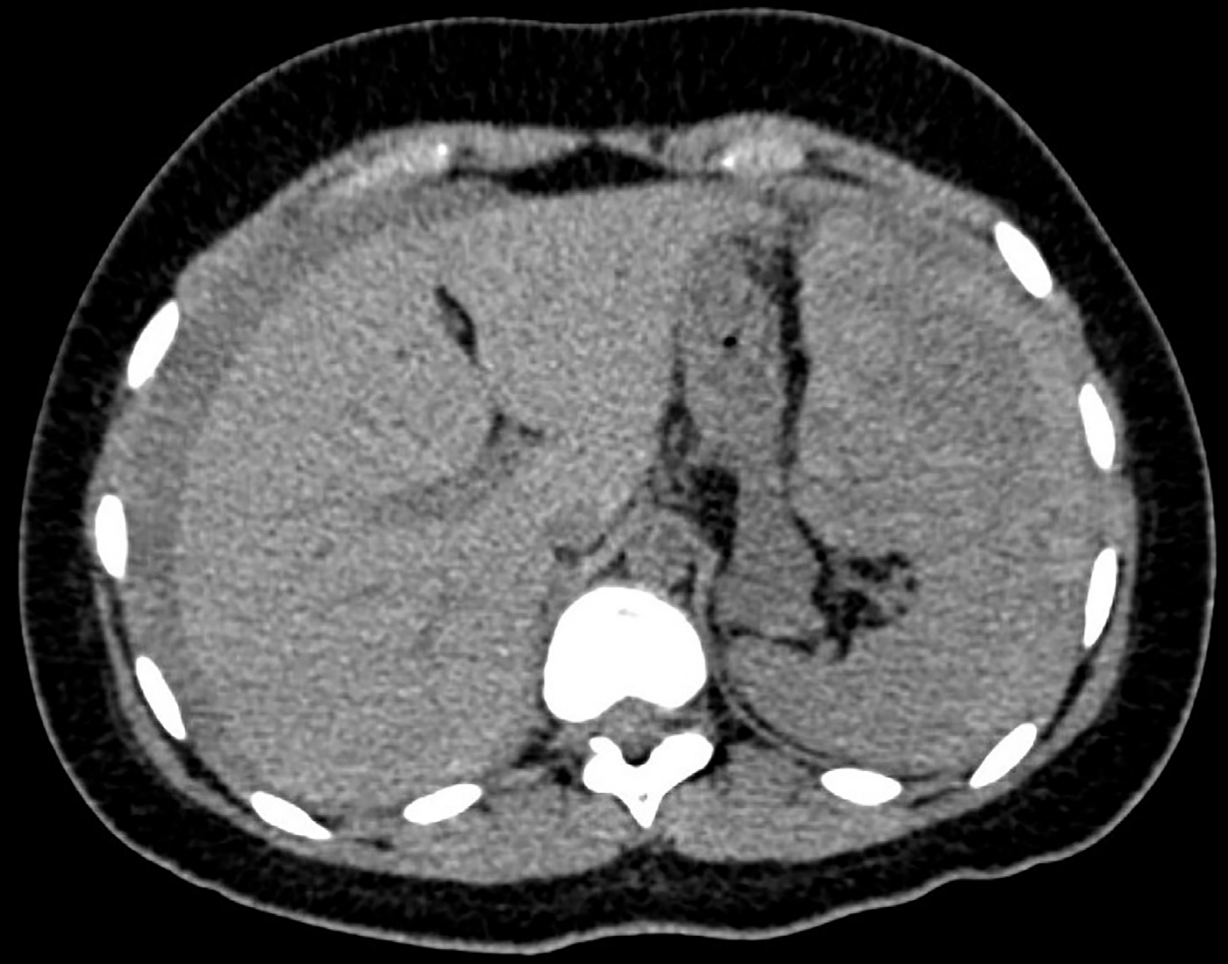
Axial view.

On initial evaluation at a local hospital, the patient was alert but in distress due to pain. Her blood pressure was mildly low and heart rate elevated (reflecting a possible evolving hemorrhagic shock). Physical examination revealed tenderness and guarding in the LUQ of the abdomen. Notably, she did not report referred left shoulder pain at that time. Laboratory studies on arrival showed a hemoglobin drop from a baseline of ∼12 g/dL to 8.5 g/dL, indicating acute blood loss. An urgent contrast-enhanced abdominal CT scan was performed, which demonstrated a splenic injury with an extensive subcapsular hematoma and free intraperitoneal fluid consistent with hemoperitoneum. There was no evidence of splenic tumors or aneurysms on the imaging, and other abdominal organs appeared normal. Given the absence of trauma and these findings, a diagnosis of spontaneous splenic rupture was made (Figs. 1 and 2).

The patient received initial resuscitation including 1 unit of packed red blood cells transfusion at the referring hospital. Because specific factor replacement (e.g. cryoprecipitate, which contains factor XIII) was not available at that facility, she was transferred to our tertiary care center for definitive management. Upon arrival, she was hemodynamically unstable, with a systolic blood pressure in the low 90s mmHg and tachycardia, despite ongoing fluid resuscitation. She was promptly taken for an emergency exploratory laparotomy.

**Fig. 3:**
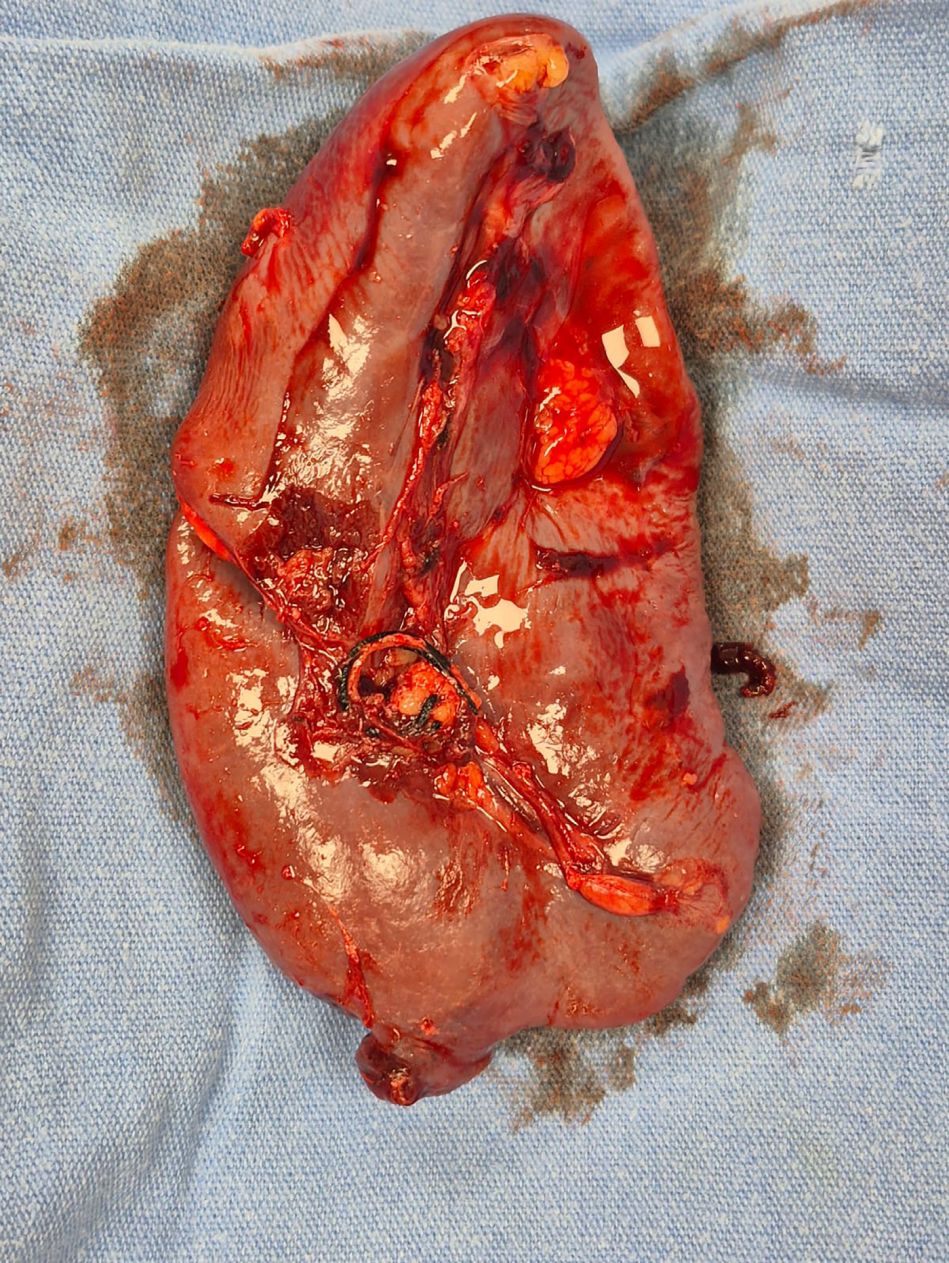
Surgical specimen, macroscopically a normal spleen is seen without involvement of the splenic hilum.

**Fig. 4:**
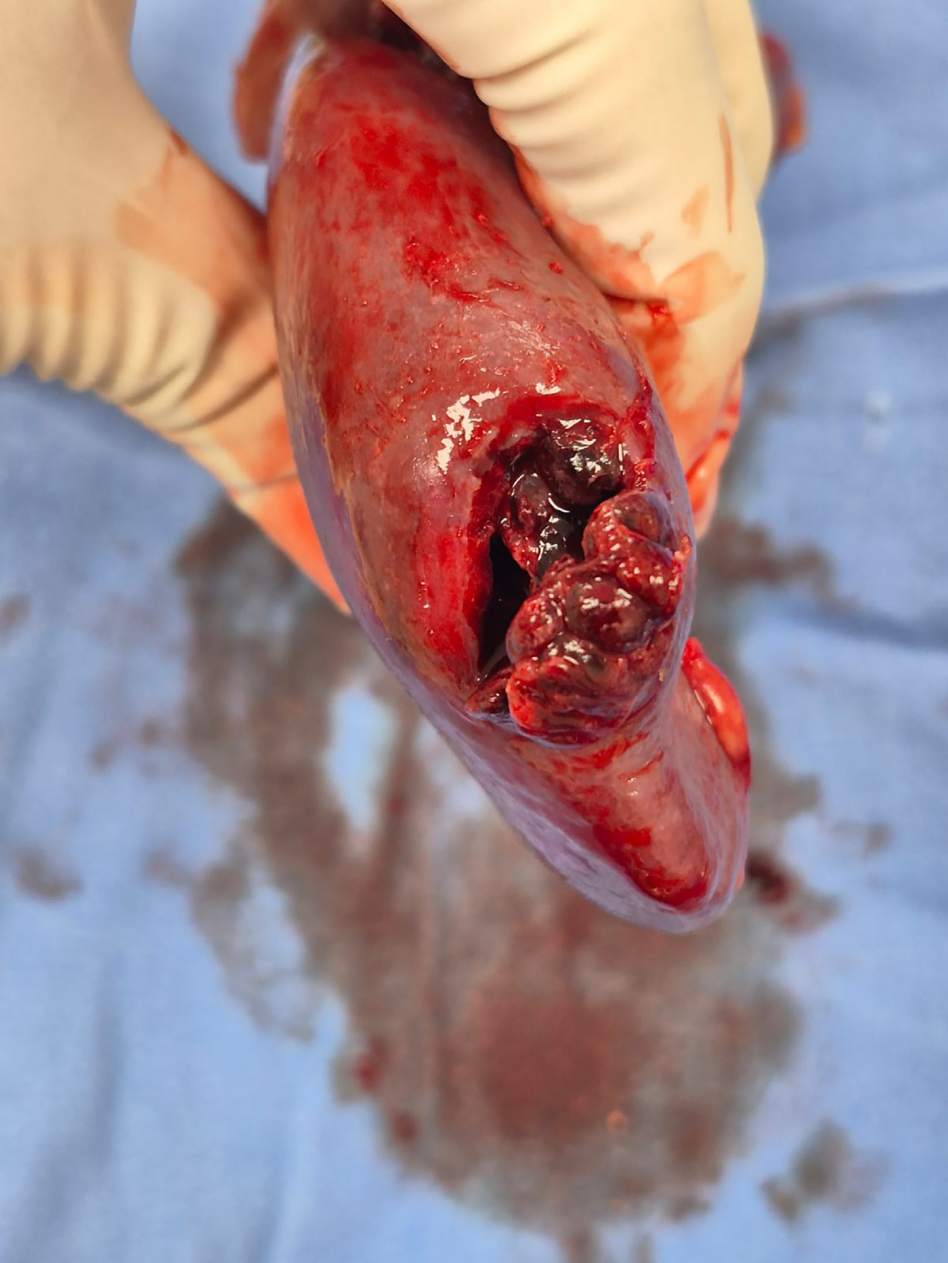
Grade 3 injury of the upper pole of the spleen.

Intraoperative findings confirmed a large amount of intraperitoneal hemorrhage (approximately 2200 mL of blood in the abdominal cavity) and a splenic laceration. The spleen had a ∼3 cm deep laceration on the inferior pole (AAST Grade III injury) with an expanding subcapsular hematoma, but no hilar vascular disruption (Fig. 3). There were no adhesions or anatomical abnormalities of the spleen noted. Active bleeding was observed from the laceration site (Fig. 4). Given the extensive hemorrhage and the patient’s underlying coagulopathy, an open splenectomy was performed. The spleen was removed in its entirety and sent for pathology. Inspection of the spleen revealed no obvious malignancy or infiltrative disease; The abdominal cavity was thoroughly evacuated of clots and irrigated. Hemostasis was achieved after spleen removal, with careful attention to drain placement.

Postoperatively, the patient was admitted to the intensive care unit and managed with a multidisciplinary approach involving surgery and hematology. She received aggressive blood product support, including cryoprecipitate transfusions (6 units every 12 hours as recommended by hematology) to replace factor XIII and other clotting factors, and additional packed red blood cells as needed. In the first 48 hours after surgery, her hemoglobin trended down to a nadir of 7.3 g/dL, but there were no signs of new active bleeding on examination or via drains (which contained only serosanguinous fluid). The hemoglobin stabilized with transfusions and factor replacement. Laboratory coagulation tests (prothrombin time and activated partial thromboplastin time) remained within normal ranges throughout, consistent with factor XIII deficiency (which typically does not prolong routine clotting assays). The patient’s abdominal pain improved significantly after surgery, and she was mobilized out of bed by postoperative day 2. By postoperative day 4, she was tolerating oral diet, with return of normal bowel function and stable vital signs. She did not develop fever or other signs of infection. Pathology of the excised spleen later confirmed a normal splenic tissue architecture with no evidence of tumor, infiltrative disease, or significant inflammation; the changes were consistent with an acute hemorrhagic rupture.

Prior to discharge, the patient was started on appropriate postsplenectomy prophylaxis, including immunizations against encapsulated organisms (pneumococcus, Haemophilus influenzae type b, and meningococcus) and a plan for lifelong antibiotic prophylaxis was put in place, given her increased risk for overwhelming postsplenectomy infection. She was discharged in stable condition on postoperative day 6. At a follow-up visit 1 month later, she had recovered well from surgery, her hemoglobin had improved with iron supplementation and continued factor XIII replacement therapy, and no further bleeding episodes had occurred.

## Discussion

This case illustrates an extremely rare event: a spontaneous splenic rupture occurring in a patient with congenital factor XIII deficiency. Our patient had no evidence of intrinsic splenic disease (such as tumor or infection), but did have a systemic bleeding predisposition due to factor XIII deficiency. Thus, her case would be classified as a pathological splenic rupture (atraumatic, but with a predisposing coagulopathy), rather than a “true idiopathic” rupture by strict criteria.

A variety of conditions have been linked to atraumatic splenic rupture. In a review, up to 93% of spontaneous splenic ruptures had at least 1 identifiable risk factor or splenic pathology, with only ∼7% being idiopathic [5]. Infections are a major category—for example, infectious mononucleosis (Epstein-Barr virus) is well known to cause splenomegaly and occasional rupture, and other viruses (hepatitis, CMV, HIV, etc.) have also been implicated. Hematologic malignancies (such as leukemias and lymphomas) account for a significant share of cases (around 13% in 1 large review), often due to splenic infiltration by malignant cells and consequent spleen enlargement or infarction. Benign tumors like hemangiomas or lymphangiomas in the spleen are rarer causes, but can lead to rupture especially if they undergo spontaneous bleeding [2].

Inherited bleeding disorders can predispose individuals to spontaneous internal hemorrhages. Hemophilia A (Factor VIII deficiency) is the most common severe congenital coagulopathy and has occasionally been associated with spontaneous splenic bleeding. Factor XIII deficiency, an inherited clotting disorder that leads to unstable fibrin clot formation, is far less common than hemophilia and has been previously reported as a cause of spontaneous splenic rupture in the literature [2]. While rare, spontaneous splenic rupture has been reported in the setting of Factor XIII deficiency. Moreover, to the best of our knowledge, recurrent spontaneous splenic rupture in a patient with Factor XIII deficiency has been documented in only 1 prior case [[2], [3], [4]].

In factor XIII deficiency, the fibrin clots formed are unstable and prone to early fibrinolysis, which means hemorrhages may continue longer than in patients with normal clot stability. This may be due to the fact that Factor XIII is the last enzyme to be activated in the blood coagulation pathway and functions to cross-link fibrin chains, stabilizing fibrin and protecting it from fibrinolysis [2]. In our patient, it is plausible that a small tear or vessel rupture in the spleen (possibly precipitated by a minor increase in intra-abdominal pressure during routine activity) led to a subcapsular hematoma that failed to stabilize, eventually causing the splenic capsule to give way. Minor physical stresses such as coughing, sneezing, or straining have been noted as possible triggers for splenic rupture in susceptible individuals. Our patient’s activity at onset (household chores) could represent such a trivial stress that, combined with her impaired clotting, resulted in hemorrhage.

The sequence of events in spontaneous splenic rupture often involves an initial subcapsular hematoma followed by delayed rupture. As described in the literature, once bleeding starts within the spleen, the capsule may initially contain it, forming a hematoma that provides a tamponade effect. If the patient’s coagulation is normal and the tear is small, this subcapsular hematoma might remain intact or even resorb [[2], [3], [4]]. However, in coagulopathic patients, the hematoma can expand rapidly and overwhelm the capsule’s capacity. In our case, the presence of factor XIII deficiency likely prevented stable clot formation, allowing the bleeding to continue unabated. The classical triad of splenic rupture is acute anemia, abdominal distension, and shock, but this triad often manifests late in the course of bleeding. Early on, patients may only have localized pain (and sometimes referred shoulder pain). Indeed, our patient presented with pain well before signs of frank peritonitis or hemodynamic collapse became evident, which is consistent with the notion that prompt imaging was crucial to diagnosis. In atraumatic cases, delay in diagnosis is common because the presentation can mimic other, more benign causes of abdominal pain, and clinicians may not immediately consider splenic rupture without a history of injury. This delay contributes to the high morbidity and mortality of spontaneous splenic rupture, as emphasized in multiple review [[2], [3], [4]]. Fortunately, in this case, the patient’s known bleeding disorder and the severity of her symptoms prompted an early imaging evaluation, which led to a timely diagnosis and intervention.

Management of spontaneous splenic rupture must be individualized, taking into account the patient’s hemodynamic stability, the extent of splenic injury, and any underlying conditions. In hemodynamically unstable patients or those with massive hemoperitoneum, emergent surgical intervention (splenectomy) is the standard, life-saving treatment in the majority of cases. Our patient had a large volume hemorrhage and instability, warranting immediate splenectomy. In some stable patients with less severe hemorrhage, especially if the bleeding risk can be mitigated (for example, by correcting a coagulopathy), nonoperative approaches may be attempted. These can include angioembolization of the splenic artery or careful observation with serial imaging and supportive care [3]. Perez et al. describe a case of spontaneous splenic rupture of unknown etiology where initial management with splenic artery embolization was tried, though the patient ultimately required surgery due to continued bleeding In cases linked to coagulation disorders, addressing the coagulopathy is critical. In the neonatal hemophilia case, aggressive factor VIII replacement allowed for avoidance of surgery [5]. In our Factor XIII deficient patient, we immediately supplemented factor XIII via cryoprecipitate, but the extent of hemorrhage still necessitated splenectomy. Bhan and Al-Hilli [4] describe 2 cases of spontaneous splenic rupture in Factor XIII deficient patients. In 1 case, the patient was managed conservatively with blood transfusions and Factor XIII cryoprecipitate, while the other required splenectomy. It is conceivable that had her bleed been smaller, factor XIII replacement might have sufficed to control it without surgery; however, given the risk of sudden rupture of a large hematoma, operative management was the safer course.

Outcomes after spontaneous splenic rupture depend on rapid diagnosis and treatment. Splenectomy, while often curative for the immediate problem, comes with the long-term consideration of asplenia. Patients require appropriate immunizations and sometimes prophylactic antibiotics to prevent overwhelming postsplenectomy infections. Our patient received the recommended vaccinations and will be monitored closely by both surgeons and hematologists moving forward. Additionally, her case highlights the importance of multidisciplinary care: the coordination between the trauma surgery team and hematology was crucial in managing both surgical hemorrhage and the underlying coagulopathy. This collaborative approach ensured she received factor XIII replacement promptly and in the postoperative period to prevent re-bleeding, analogous to the management reported in other coagulopathic patients.

Finally, this case adds to the existing literature by presenting an additional instance of spontaneous splenic rupture in the setting of Factor XIII deficiency, a rare association previously documented in a limited number of reports [[2], [3], [4]]. It underscores that even rarer bleeding disorders can precipitate life-threatening abdominal emergencies. Clinicians should be aware that a complaint of left upper abdominal pain in a patient with a known coagulopathy (even without trauma) could represent an internal hemorrhage, and timely imaging is warranted. Likewise, when an atraumatic splenic rupture is encountered, a thorough workup for underlying causes is essential—including evaluation for infections, malignancy, and coagulation abnormalities—even if initial surveys (such as viral panels or imaging) are unrevealing. In our patient, the known factor XIII disorder provided an immediate explanation, but if it were not known, diagnostic evaluation for a bleeding diathesis would have been indicated given the intraoperative finding of diffuse bleeding. Identifying an underlying cause not only helps tailor acute management (e.g., giving appropriate factor replacement) but also guides long-term therapy to prevent recurrence.

## Conclusion

Spontaneous rupture of the spleen is a rare surgical emergency that can occur even in young patients with no history of trauma. This case demonstrates that congenital factor XIII deficiency, a rare coagulation disorder, can lead to spontaneous life-threatening hemorrhage. The patient’s outcome highlights several key points. First, spontaneous splenic rupture should be considered in the differential diagnosis of acute abdominal pain, especially in individuals with known bleeding tendencies. Early imaging and prompt surgical consultation can be life-saving. Second, the presence of a coagulation disorder such as factor XIII deficiency can exacerbate hemorrhage; thus, concurrent hematologic management (with appropriate factor replacement) is essential to optimize outcomes. Third, definitive treatment in unstable patients remains splenectomy in most instances, and this carries the need for vigilant long-term preventive care (vaccinations and possibly antibiotic prophylaxis) to mitigate postsplenectomy risks.

In summary, we report another instance of spontaneous splenic rupture occurring in the setting of Factor XIII deficiency, further emphasizing the clinical significance of congenital coagulopathies as rare yet critical risk factors for atraumatic internal hemorrhage. Drawing from this case and the compiled literature, we reinforce the importance of early recognition of spontaneous splenic rupture, a comprehensive understanding of its multifactorial etiology (including underlying coagulation disorders), and a management approach combining timely surgical intervention with targeted medical therapy. A combination of heightened awareness and a high index of suspicion among clinicians is needed to improve diagnostic timeliness and patient outcomes in such unusual yet perilous presentations

## Patient consent

Complete written informed consent was obtained from the patient for the publication of this study and accompanying images.
